# Total Phenolic and Total Flavonoid Content, Individual Phenolic Compounds and Antioxidant Activity in Sweet Rowanberry Cultivars

**DOI:** 10.3390/antiox12040913

**Published:** 2023-04-12

**Authors:** Jana Orsavová, Tunde Juríková, Růžena Bednaříková, Jiří Mlček

**Affiliations:** 1 Language Centre, Faculty of Humanities, Tomas Bata University in Zlín, Štefánikova 5670, 760 01 Zlín, Czech Republic; orsavova@utb.cz; 2 Institute for Teacher Training, Faculty of Central European Studies, Constantine the Philosopher University in Nitra, 949 74 Nitra, Slovakia; tjurikova@ukf.sk; 3 Department of Food Analysis and Chemistry, Faculty of Technology, Tomas Bata University in Zlín, 760 01 Zlín, Czech Republic; rvaskova@utb.cz

**Keywords:** rowanberry, cultivars, polyphenolic compounds, antioxidant activity, correlation analyses

## Abstract

Sweet rowanberry and its cultivars represent a less-known fruit species with significant antioxidant activity, mostly promoted by polyphenolic compounds. This paper examined seven *Sorbus* cultivars and evaluated their total polyphenolic and flavonoid content, as well as the content of individual polyphenolic compounds from the group of phenolic acids and flavonoids. It also determined their antioxidant activity using DPPH, ACW and ACL. Furthermore, to reflect the distribution of the contribution to antioxidant activity, correlations between antioxidant activity and the contents of ascorbic acid, vitamin E and individual phenolic compounds were established. The highest total phenolic content of 8307.4 mg kg^−1^ was determined in ‘Granatina’, with the main contribution of phenolic acid content of 7001.7 mg kg^−1^ and a significantly lower total flavonoid content of 1304.6 mg kg^−1^. Flavanols represented the most abundant group of flavonoids, with catechin being the second most frequent flavanol with the highest content of 633.67 mg kg^−1^ in ‘Granatina’. Flavonols were represented by rutin and quercetin. ‘Businka’ displayed a significant vitamin E content of 4.77 mg kg^−1^, and ‘Alaja Krupnaja’ had the highest vitamin C level of 7.89 g kg^−1^. These results emphasize their potential health and nutritional benefits and, thus, their promising and valuable role in the food processing industry.

## 1. Introduction

The genus *Sorbus* includes five diploid species in Europe, namely *Sorbus aria*, *Sorbus aucuparia*, *Sorbus torminalis*, *Sorbus chamaemespilus* and *Sorbus domestica* [1]. Such diversity stems from interspecific hybridizations [2]. Sweet rowanberry cultivars have been evolved from rowanberries (*Sorbus aucuparia* L.) and hybrids of rowanberry with *Malus*, *Pyrus*, *Aronia* or *Mespilus* [3]. Even though the fruits of wild rowanberry excel with their high nutritional values, they have not been recommended for direct consumption due to their specific astringent taste caused mainly by tannins [4]. The first known attempts to select new sweet rowanberry cultivars (‘Rossica’ and ‘Rosina’) without astringency and bitterness date back to the 19th century in the Sudeten Mountains, a current territory of the Czech Republic. A breeding program of sweet rowanberries suitable and adaptable for the northern conditions, specifically winter hardiness, was commenced by Michrin in Russia at the beginning of the 20th century. This program introduced new hybrids of rowanberry with the *Aronia*, *Malus*, *Mespilus* and *Pyrus* species [3]. The most popular cultivars from Russia are ‘Burka’, ‘Likjornaja’, ‘Dessertnaja’, ‘Granatnaja’, ‘Rubinovaja’ and ‘Titan’ [5]. In comparison with wild species, ‘Granatnaja’ and ‘Alaja Krupnaja’ are characterized by an enhanced taste without the traces of astringency and by larger, darker fruits [6]. The most famous hybrids of *S. aucuparia* developed in Western Europe include ‘Apricot Queen’, ‘Brilliant Yellow’, ‘Chamois Glow’, ‘Pink Queen’ and ‘Salmon Queen’ [4,7].

Sweet rowanberry fruits represent a significant source of bioactive compounds, particularly polyphenolic compounds and ascorbic acid, resulting in significant levels of antioxidant activity [6]. Considerably high levels of flavonoids (quercetin derivatives rutin, hyperoside and isoquercitrin), phenolic acids (chlorogenic, neochlorogenic and caffeic acids) and proanthocyanidins have already been proven by several authors [8,9,10,11]. Caffeoylquinic acids and flavonoids, together with proanthocyanidins, have been reported by Sarv et al. (2020) as the most abundant polyphenolic substances [4]. The total phenolic content (TPC) represents 550–1014 mg 100 g^−1^ FW, anthocyanins 6–80 mg 100 g^−1^, chlorogenic acid 29–160 mg 100 g^−1^ and neochlorogenic acid 34–104 mg 100 g^−1^ FW, depending on the cultivar [3]. The content of total phenolic acids as aglycones in sweet rowanberry reached 103 mg 100 g^−1^ FW [12]. Juríková et al. (2014) determined the highest values of phenolic and flavonoid content in fruits of ‘Likernaja’ in the amounts of 5.82 g of gallic acid kg^−1^ FW and 3.85 ± 0.18 g of rutin kg^−1^ FW, respectively. Furthermore, this cultivar also showed the highest content of chlorogenic acid and quercetin, with values of 100.8 mg 100 g^−1^ FW and 51.4 mg 100 g^−1^ FW, respectively [13].

Sweet rowanberry cultivars seem to be easily grown in severe climatic and soil conditions, and what is more, they represent a valuable source of fruits with exceptional nutritional value and declared health-promoting effects. They have been used in the cosmetics, natural medicine and food industry, where they may find applications as innovative food ingredients, functional foods and nutraceuticals. Nowadays, these fruits are widely utilized in the treatment of bacterial, viral and inflammatory diseases, including tumors, diabetes and neurological and cardiovascular disorders [4]. In some countries, they have also been employed in the treatment of intestinal obstructions and different liver and gallbladder disorders [14]. Additionally, the phenolic extracts of sweet rowanberry cultivars display antibacterial effects against *Staphylococcus aureus* and specifically, the cultivar ‘Zoltnaja’ has been reported to perform an inhibitory effect on *Salmonella* and *Escherichia coli* [8]. Moreover, they are considered to be exceptional for their application in jams and jellies owing to their quality, acidity, color, general high pectin content, flavor and aroma [15].

However, due to the great diversity of the fruits of sweet rowanberry cultivars, their polyphenolic profiles and antioxidant activity levels still remain to be fully determined. Therefore, this paper aims to evaluate the chemical composition of the fruits of the selected *Sorbus* cultivars in detail and thus provide valuable information not only for the producers but for the consumers as well.

## 2. Materials and Methods

### 2.1. Fruit Samples

The experiment samples included the fruit of the following sweet rowanberry cultivars: ‘Alaja Krupnaja’, ‘Granatnaja’, ‘Granatina’, ‘Businka’, ‘Koncentra’ and ‘Discolor’. Their origin, breeding background and description are provided in Table 1. When compared with wild species, the selected *Sorbus* cultivars are characterized by an enhanced taste without any astringency or bitterness and by the large size of their fruits. Berries of the tested cultivars were collected from a minimum of five plants in the amount of 500 g per cultivar in a fully ripe state. First, fresh berries were homogenized using a blender (Bosch MSM67170, Bosh GmbH, Stuttgart, Germany) and deep-frozen in an ultra-low temperature freezer (ULUF P610 GG—Arctiko, Esbjerk, Denmark) at −80 °C for at least 24 h. Afterwards, they were lyophilized by Alpha 1–4 LSC (Christ Gefriertocknungsanlagen Gmb, Osterode am Harz, Germany) at −55 °C and 0.120 Mbar for 48 h. Lyophilized samples were homogenized and maintained in polyethylene bags equipped with a zip at −20 °C until they were subjected to the analysis.

### 2.2. Experimental Area

The experimental area owned by the Mendel University in Brno is in the cadastral area of Žabčice at a latitude of 185 m a.s.l. with the GPS coordinates 49.011598 N and 16.602572 E. It is characterized by typical continental climatic conditions, with a long-term average annual temperature of 9.2 °C and a precipitation of 519.0 mm. In 2014, the average annual temperature in Žabčice was 11.2 °C, and the precipitation was 576.7 mm. During the ripening period from May to July 2014, the average temperature was 18.3 °C, and the precipitation value was 191.2 mm. Table 2 displays long-term average values and annual temperature and precipitation values for 2014 in this experimental area.

Fruits were harvested in 2014. This year was characterized by above-average rainfall levels and higher temperatures; specifically, the average temperature exceeded the long-term average by 2 °C, and the precipitation level was 97 mm higher than the long-term average amount. The distribution of rainfall was irregular: from January to April, precipitation was below average; during July, August and September, it was well above average and accounted for more than half of the annual total amount [16].

### 2.3. Chemicals and Reagents

The chemicals ethanol, methanol and acetic acid were obtained from Penta (Prague, Czech Republic) and methanol–HPLC from LabScan (Sowińskiego, Poland). Phenolic compound standards of HPLC grade were acquired from Sigma Aldrich (St. Louis, MO, USA). Standards of ascorbic acid and D–α–tocopherol succinate were acquired from AccuStandard (New Haven, CT, USA). Further used chemicals were of analytical grade and purchased from Sigma Aldrich (St. Louis, MO, USA).

### 2.4. Extraction Methods

The extractions were conducted according to the protocols published by Orsavová et al. (2019) and Sytařová et al. (2020) [17,18]. Lyophilized fruit samples of 0.5 g were extracted in 10 mL of the extraction solution of water and methanol (70/30, *v*/*v*) to determine total phenolic content, total flavonoid content and antioxidant activity (by DPPH method). To prepare the extracts for HPLC assay, the same amount of 0.5 g of lyophilized fruit samples was extracted in a mixture of redistilled water/methanol/acetic acid (69/30/1, *v*/*v*/*v*) using screw-cap test tubes in a water bath (Memmert GmbH + Co.KG, Schwabach, Germany) and shook at 50 °C for 60 min. The extracts were then centrifuged at 2430× *g* for 15 min (Velocity 13µ, Dynamica Scientific Ltd., Milton Keynes, UK) at room temperature. Further, the extracts for vitamin C determination were prepared from 0.5 g of lyophilized fruits using 2.5 mL of a mobile phase (methanol/H_3_PO_4_/redistilled water (99/0.5/0.5, *v*/*v*/*v*)) in screw-cap test tubes in a shaker LT 2 (Kavalier, Sázava, Czech Republic) for 10 min in the dark. The extracts were then added into 10 mL volumetric flasks and filled with a mobile phase. Similarly, the extracts for vitamin E determination were prepared from 1.0 g of lyophilized fruit samples using 2.5 mL of methanol in screw-cap test tubes in an ultrasonic bath PS 04,000 A (Notus–Powersonic, Vráble, Slovakia) at 40 °C for 60 min. Afterwards, the extracts were added into 10 mL volumetric flasks and filled with methanol. Redistilled water was obtained by PURELAB Classic (ELGA, Lane End Business Park, High Wycombe, UK). The extraction for the analysis of total anthocyanin content was performed according to the protocol reported by Orsavová et al. (2019) [17]. Briefly, lyophilized fruit samples of 1.5 g were extracted in 5 mL of a mixture of methanol/water/acetic acid (70/29/1, *v*/*v*/*v*) in screw-cap test tubes in a shaking water bath (Memmert GmbH + Co.KG, Schwabach, Germany) at 50 °C for 60 min and subsequently in an ultrasonic bath at 40 °C for 60 min. The extracts were centrifuged at 3280× *g* for 15 min (Velocity 13µ, Dynamica Scientific Ltd., Milton Keynes, UK) at room temperature. Prior to the analyses, all extracts and supernatants were filtered using nylon microfilters (SYRINGE, Cronus Syringe Filter, Nylon 13 mm × 0.45 µm, Labicom, Olomouc, Czech Republic).

### 2.5. Analysis of Total Phenolic (TPC) and Total Flavonoid (TFC) Content

According to protocols by Orsavová et al. (2019), total phenolic content (TPC) was established employing the Folin–Ciocalteu method, and total flavonoid content (TFC) applying NaNO_2_, AlCl_3_·6H_2_O and NaOH using a UV/VIS spectrometer Lambda 25 (PerkinElmer, Waltham, MA, USA) [17]. The results were expressed as grams of gallic acid equivalent per kg (g GA kg^−1^ DW) for TPC and as grams of rutin equivalent per kg (g RE kg^−1^ DW) for TFC.

### 2.6. Analysis of Total Anthocyanin Content (AC)

Total anthocyanin content (AC) was established by pH differential absorbance method (AOAC official method 2005.02) employing a UV/VIS spectrometer Lambda 25 (PerkinElmer, Waltham, MA, USA) [19] and following the protocol by Lee et al. (2017). The results were expressed as grams of cyanidin-3-*O*-glucoside equivalent (g COG kg^−1^ DW; molecular weight = 449.2 g mol^−1^, molar extinction coefficient = 26,900 L cm^−1^ mol^−1^).

### 2.7. Analysis of Vitamins C and E

The contents of vitamins C and E were recorded following the protocols reported by Orsavová et al. (2019) and Sytařová et al. (2020). The analysis employed an HPLC system, UltiMate^®^ 3000 (Dionex, Sunnyvale, CA, USA), equipped with a diode-array detector [17,18]. For the vitamin C analysis, the reverse-phase column Acclaim 120 C8 (Dionex, MA, USA) with dimensions of 150 × 2.1 mm and a particle size of 5 µm was applied. The mixture of methanol/H_3_PO_4_/r–H_2_O was used in a ratio of 99:0.5:0.5 (*v*/*v*/*v*) as a mobile phase in an isocratic mode, and the flow rate was set to 0.8 mL min^−1^ with an injection volume of 20 µL and column temperature of 25 °C maintained throughout the 10 min analysis. Chromatograms were registered at 275 nm. For the analysis of vitamin E, the column Kinetex C-18 (Phenomenex, Torrance, CA, USA) with dimensions of 150 × 4.6 mm and a particle size of 2.6 µm was applied. A mixture of methanol (HPLC)/r–H2O was used in a ratio of 95:5 (*v*/*v*) as a mobile phase in an isocratic mode, and the flow rate was set to 1 mL min^−1^ with an injection volume of 20 µL and column temperature of 30 °C maintained throughout the 20 min analysis. Chromatograms were monitored at 230 nm. Then, vitamin C and E contents were calculated using the calibration curves employing ascorbic acid and D–alpha–tocopherol succinate as standards. The contents of vitamin C were expressed in g kg^−1^ DW and those of vitamin E in mg kg^−1^ DW.

### 2.8. Determination of Phenolic Compounds Using HPLC

Individual phenolic compounds were determined using an HPLC device, UltiMate^®^ 3000 (Dionex, Sunnyvale, CA, USA), equipped with a diode-array detector and Kinetex column C-18 (Phenomenex, Torrance, CA, USA), following the procedure described by Orsavová et al. (2019) [17]. Solvent A was a mixture of water/acetic acid prepared in a ratio of 99:1 (*v*/*v*) and solvent B consisted of water/acetonitrile/acetic acid in a ratio of 67:32:1 (*v*/*v*/*v*), with the gradient mode set as follows: 0–10 min: 90% A + 10% B; 10–16 min: 80% A + 20% B; 16–20 min: 60% A + 40% B; 20–25 min: 50% A + 50% B; 25–27 min: 60% A + 40% B; 27–35 min: 90% A + 10% B. The flow rate was set to 1 mL min^−1^ with an injection volume of 10 µL and column temperature of 23 °C maintained throughout the 35 min analysis. Chromatograms were recorded at 275 nm. Individual phenolic compounds were identified by the retention times and the method of standard addition. Data signals were processed by LC ChromeleonTM 7.2 Chromatography Data System (Dionex, Sunnyvale, CA, USA).

### 2.9. Determination of Antioxidant Activity Using DPPH and PCL

Antioxidant activity (AOA) was examined following the protocol published by Orsavová et al. (2019) [17] employing DPPH (2,2-diphenyl-1-picrylhydrazyl; Sigma Aldrich, MO, USA) and PCL. During DPPH analysis, the absorbance was recorded at 515 nm by Lambda 25 (PerkinElmer, Waltham, MA, USA). Trolox, obtained at Sigma Aldrich, MO, USA, was used as a standard. The results were expressed as grams of Trolox equivalent per kg (g Trolox kg^−1^ DW). For the PCL analysis, official ACW and ACL protocols were applied using ACW and ACL kits (Analytik Jena AG, Jena, Germany) for water-soluble and lipid-soluble compounds, respectively, in PHOTOCHEM (Analytik Jena AG, Jena, Germany). ACW and ACL were quantified by applying calibration curves. Ascorbic acid was used as a standard for ACW and Trolox for ACL, with the results expressed as grams of ascorbic acid equivalent per kg (g AA kg^−1^ DW) or Trolox equivalent (g Trolox kg^−1^ DW).

### 2.10. Statistical Analysis

All experiments were repeated three times, and their results were expressed as means and standard deviations. SPSS 12.0 (SPSS Inc., Chicago, IL, USA) was applied to confirm significant differences between the examined values. The Shapiro–Wilk test was used to test normal data distribution. Then, for normally distributed data, one-way analysis of variance (Anova, Tukey’s test) was employed with the level of significance set to *p* < 0.05. For abnormal data distribution, a non-parametric Kruskal–Wallis test was used with the same significance level. Pearson correlation coefficients (R) were calculated using Microsoft Office Excel 2013 (Redmond, WA, USA), and Evans’ classification was applied to assess the strength of correlations [20].

## 3. Results

### 3.1. Determination of Total Polyphenolic (TPC), Total Flavonoid (TFC) and Anthocyanin Content (AC)

The values of total polyphenolic (TPC), total flavonoid (TFC) and anthocyanin content (AC) in the examined samples of sweet rowanberry significantly differed between the cultivars, as can be seen in Table 3.

TPC ranged from 8.81 g GA kg^−1^ in ‘Granatnaja’ to 16.31 GA kg^−1^ in ‘Businka’, which is in accordance with a previously published study by Šavikin et al. (2017) examining 26 cultivars of sweet rowanberry from Montenegro with TPC ranging from 5.25 g GA kg^−1^ to 15.91 g GA kg^−1^ [10].

Sarv et al. (2021) studied TPC values in 16 sweet rowanberry cultivars, monitoring values ranging between 2.53 mg GA g^−1^ in ‘Krasnaja’ and 15.05 mg GA g^−1^ DW in ‘Likernaja’ [21]. Lower TPC values were determined in ‘Alaja Krupnaja’ (6.46 ± 0 g GA kg^−1^) and ‘Bussinka’ (2.88 ± 0 g GA kg^−1^) in comparison with the results of this study, which determined TPC in the amounts of 9.00 ± 0.02 g GA kg^−1^ in ‘Titan’. This is in alignment with the data recorded by Hukkinen et al. (2006), who determined TPC in the amounts of 8.08 ± 1.9 g GA kg^−1^ [3].

On the other hand, significantly lower TPC values were reported by Jabłońska–Ryś et al. (2009) when they studied samples of sweet rowanberry from Poland [22]. Similarly, in the cultivar ‘Granatnaja’ from the Czech Republic, lower TPC values of 3.65 g GA kg^−1^ FW were recorded in 2011 and 2012 [12].

A comparable TPC content of 8.19 g GA kg^−1^ FW was established in ‘Granatnaja’ from the Czech Republic in 2011 and 2012. The lower values of 8.11 g GA kg^−1^ FW in ‘Granatina’ and 6.28 g GA kg^−1^ FW in ‘Titan’ proved the generally variable composition of sweet rowanberry fruits [6]. The total phenolic content in *Sorbus* has generally shown a strong dependence on the maturity stage of the berries, while the recovery of phenolics has been influenced by the extraction solvent [23].

This study evaluated the significant differences in total flavonoid content (TFC) among all assayed cultivars. The lowest content of 15.04 g RU kg^−1^ was monitored in ‘Koncentra’, in contrast to the highest values of 26.85 g RU kg^−1^ in ‘Granatina’ and 26.69 g RU kg^−1^ in ‘Granatnaja’.

Severalfold lower TFC values were reported in three cultivars from the Czech Republic in 2011 and 2012: 5.35 g GA kg^−1^ FW in ‘Granatnaja’, 5.65 g GA kg^−1^ FW in ‘Granatina’ and 4.70 g GA kg^−1^ FW in ‘Titan’ [6].

Very low TFC values of 2.55 g RU kg^−1^ FW were reported in ‘Granatnaja’ from the Czech Republic by Juriková et al. (2014) [12]. Kivrak et al. (2014) determined 18.56 g quercetin kg^−1^ of flavonoids in wild berries of *Sorbus umbellata* from Turkey [24].

Anthocyanins represented the second most abundant group of polyphenols in sweet rowanberry cultivars. According to the study by Sarv et al. (2021), ‘Burka’ had a considerable total anthocyanin content of 7.27 mg g^−1^ DW and ‘Granatnaja’ had the lowest one at 3.20 mg g^−1^ DW [21]. Paulovicsova et al. (2009) identified an anthocyanin content of 0.34 ± 0.099 mg kg^−1^ DW in the fruits of the cultivar ‘Sladkoploda Moravska’ [25].

The results of this experiment have clarified statistically significant differences in anthocyanin content (AC). Light-colored ‘Alaja Krupnaja’ displayed a very low anthocyanin content at 1.19 mg COG 100 g^−1^, together with ‘Koncentra’ and ‘Discolor’, which had 5.36 mg COG 100 g^−1^ and 5.50 mg COG 100 g^−1^, respectively. In contrast, the highest values of 51.38 mg COG 100 g^−1^ and 50.20 mg COG 100 g^−1^ were recorded in ‘Granatnaja’ and ‘Titan’, respectively.

According to Zymone et al. (2018), powders from ‘Burka’ and ‘Likernaja’ fruit contained the highest anthocyanin contents [26].

More than double the anthocyanin content was reported by Hukkanen et al. (2006) in ‘Granatnaja’ (116.8 mg COG 100 g^−1^ FW) and ‘Titan’ (101.6 mg COG 100 g^−1^ FW) from Finland [3].

### 3.2. Determination of Individual Phenolic Compounds by HPLC

#### 3.2.1. Total Contents of Phenolic Compounds by RP–HPLC

**Figure 1:**
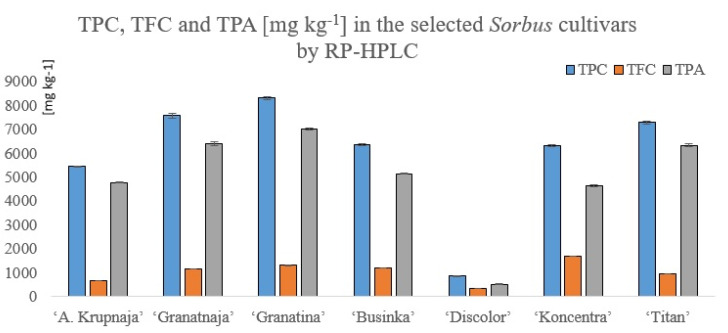
Total phenolic (TPC), flavonoid (TFC) and phenolic acid (TPA) contents in [mg kg^−1^] in the selected *Sorbus* cultivars detected by RP–HPLC.

The total phenolic (TPC), total flavonoid (TFC) and total phenolic acid contents (TPA) are listed in Figure 1. For TPC, significant differences were determined by RP–HPLC. The highest TPC content of 8307.4 mg kg^−1^ was recorded in the cultivar ‘Granatina’. Further significant TPC levels were identified in ‘Granatnaja’ (7568.0 mg kg^−1^) and ‘Titan’ (7290.5 mg kg^−1^). Comparable values of 6350.0 mg kg^−1^ and 6332.2 mg kg^−1^ were determined in ‘Businka’ and ‘Koncentra’, respectively. On the other hand, a contrastingly low content of 857.4 mg kg^−1^ was established in ‘Discolor’. The content of phenolic acids mainly contributing to TPC ranged from 524.7 mg kg^−1^ in ‘Discolor’ to 7001.7 mg kg^−1^ in ‘Granatina’. The total flavonoid content (TFC) ranged between 524.7 mg kg^−1^ in ‘Discolor’ and 1304.6 mg kg^−1^ in ‘Granatina’.

According to Kampuss et al. (2009), the total phenolic content ranged between 162 mg 100 g^−1^ of fresh weight in ‘Krasnaja Krupna’ and 485 mg 100 g^−1^ in ‘Likernaja’ in samples from Latvia [27].

In the studies by Mattila et al. (2006), the average TPC of 24 analyzed sweet rowanberry fruit samples reached the highest value of 103 mg 100 g^−1^ FW [11]. In all the assayed cultivars, TPA ranged from 524.7 mg kg^−1^ in ‘Discolor’ to 7001.7 mg kg^−1^ in ‘Granatina’, which significantly contributed to TPC. The study by Mattila et al. (2006) comparing the phenolic profiles of berries established an average phenolic acid content of 75 mg 100 g^−1^. In this study, the total flavonoid content ranged from 332 mg kg^−1^ in ‘Discolor’ to 1304.6 mg kg^−1^ in ‘Granatina’ [11].

#### 3.2.2. Individual Phenolic Compounds by RP–HPLC

Phenolic acids have been observed as the most abundant phenolic substances in *Sorbus*. On the other hand, flavonoids have shown lower contents [28].

As the major flavonoids, quercetin, isoquercetin, kaempferol, rutin, hyperoside and isorhamnetin were reported in the examined samples of fruits, leaves and inflorescences of the selected *Sorbus* cultivars [4].

**Figure 2:**
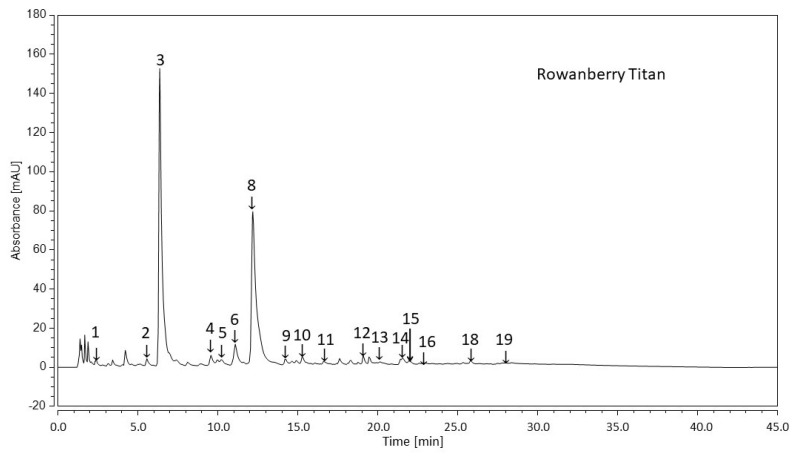
The profile of polyphenolic compounds in sweet rowanberry ‘Titan’: gallic acid (1), protocatechuic acid (2), neochlorogenic acid (3), 4–hydroxybenzoic acid (4), epigallocatechin (5), catechin (6), vanillic acid (7), chlorogenic acid (8), caffeic acid (9), syringic acid (10), epicatechin (11), p–cumaric acid (12), ferulic acid (13), sinapic acid (14), ellagic acid (15), rutin (16), t–cinnamic acid (17), protocatechuic acid ethyl ester (18) and resveratrol (19).

The contents of individual flavonoids, stilbenes and resveratrol, together with the sum of total flavonols (TFLOC) and flavanols (TFLAC) are provided in Table 4 and Figure 2.

Flavonols were represented mostly by rutin, with amounts ranging from 9.8 mg kg^−1^ in ‘Koncentra’ and 10.6 mg kg^−1^ in ‘Titan’ to 71.1 mg kg^−1^ in ‘Alaja Krupnaja’. Several studies declared various contents of total flavonols depending on the cultivars. In the samples of sweet rowanberry from Lithuania, Raudonis (2014) established rutin in the amount of 90.0 mg kg^−1^ [29]. Higher concentrations were detected in ‘Alaja Krupnaja’ with an amount of 97 mg kg^−1^, ‘Granatnaja’ with 51 mg kg^−1^, ‘Businka’ with 23 mg kg^−1^, ‘Koncentra’ with 24 mg kg^−1^ and, lastly, ‘Titan’ with 74 mg kg^−1^, all from Lithuania as well [26]. A less significant concentration of 1.81 mg kg^−1^ was found in *Sorbus umbellata* fruits from Turkey [24], in contrast with the results of Jurikova et al. (2014), who reported 759.5 mg kg^−1^ FW of rutin in ‘Granatina’ from the Czech Republic in 2012 and 2013 [12]. A considerable variability of rutin contents ranging from 36.9 mg kg^−1^ to 598.3 mg kg^−1^ was identified in 26 cultivars of sweet rowanberry from Serbia and Montenegro [10].

Quercetin was detected only in the cultivar of ‘Alaja Krupnaja’ in the amount of 2.4 mg kg^−1^ which is in contrast with its amount in the 26 cultivars from Serbia and Montenegro ranging from 2.8 mg kg^−1^ to 83.5 mg kg^−1^ [10] and also its content of 1.30 mg kg^−1^ found in *Sorbus umbellata* from Turkey [24]. A very high quercetin content of 440.3 mg kg^−1^ was reported in the samples of ‘Granatina’ from the Czech Republic in 2012 and 2013 by Juriková et al. (2014) [12]. Kaempferol was not detected in the examined cultivars; however, Kıvrak et al. (2014) determined 0.22 mg kg^−1^ in the samples of *Sorbus umbellata* [24].

The quercetin content in the fruits of *S. aucuparia*, *S. intermedia* and *S. aria* was 0.51, 0.31 and 0.09 mg g^−1^, respectively. Kaempferol was quantified in the fruits, leaves and inflorescences of the same *Sorbus* species, with its highest content recorded in *S. aucuparia* [30].

When compared with the results of this study, a significantly higher value of 1290 mg kg^−1^ of total flavonols was found in ‘Granatnaja’ from Finland by Kylli et al. (2010).

The total flavanol content represented a significant proportion of flavonoids and was present in amounts ranging from 298.8 mg kg^−1^ in ‘Discolor’ to 1668.6 mg kg^−1^ in ‘Koncentra’.

Epigallocatechin (EGC) was the predominant flavanol in four of the analyzed cultivars, with the highest amount of 1167.5 mg kg^−1^ in ‘Koncentra’; the rest of the samples showed amounts ranging from 244.3 mg kg^−1^ in ‘Discolor’ to 625.4 mg kg^−1^ in ‘Businka’. The catechin (CA) content ranged from 23.4 mg kg^−1^ in ‘Discolor’ to 633.6 mg kg^−1^ in ‘Granatina’, while epicatechin (EC) was present in smaller amounts (from 3.3 mg kg^−1^ in ‘Koncentra’ to 31.1 mg kg^−1^ in ‘Discolor’). A significantly lower EC concentration of 0.38 mg kg^−1^ was identified in the samples of *Sorbus umbellata* from Turkey [24], in contrast to a very high amount of 862.50 mg kg^−1^ recorded in the samples from the Czech Republic in 2008 and 2010 [31].

Stilbene resveratrol (RES) was established only in very low amounts. The highest content of 3.3 mg kg^−1^ was determined in ‘Alaja Krupnaja’ and the lowest of 0.5 mg kg^−1^ in ‘Titan’. It was not identified in the cultivar ‘Discolor’, similarly to the *Sorbus umbellata* samples from Turkey [24].

A significant diversity in the profile of flavonols has been observed depending on the particular cultivar (Table 4, Figure 2). All rowanberry fruit powder samples showed the presence of rutin, hyperoside and isoquercitrin [26].

### 3.3. Determination of Phenolic Acids

The greatest amounts of phenolic acids were found in rowanberries by Mattila et al. (2006), in contrast with their amounts in the samples of chokeberry, saskatoon berry, blueberry, raspberry, bilberry, cloudberry, rosehip, lingonberry, black currant and bog whortleberry [11]. Mrkonjic et al. (2019) used LC–MS/MS to determine 15 phenolic acids, with the predominance of chlorogenic acid in the fruits of *S. domestica* [32].

Chlorogenic (3-*O*-caffeoylquinic acid, 3–CQA) and neochlorogenic (5-*O*-caffeoylquinic acid, 5–CQA) acids represent the most abundant phenolic acids [10], accounting for 56–80% of the total phenolic content in *Sorbus* berries [8]. Cinnamic, vanillic, p–coumaric and benzoic acids were detected only in trace amounts in the fruits of *S. aucuparia* [33] and *S. domestica* [34]; p–coumaric acid was determined in *S. discolor* fruits as well [35]. Raudonis et al. (2014) established a significant variability in the contents of phenolic acid and flavonoid substances and the levels of antioxidant activity in the fruits of the *Sorbus* species, which is in accordance with the results of this study [29].

This study has identified the presence of derivatives of benzoic acid (DBA) (gallic acid (GA), vanillic acid (VA), syringic acid (SI), protocatechuic acid (PC), protocatechuic acid ethyl ester (PCEE), 4–hydroxybenzoic acid (HB) and ellagic acid (EL)) and the group of derivates of cinnamic acid (DCA) (t–cinnamic acid (TCA), hydroxycinnamic acid (HCA), coffeic acid (CA), ferullic acid (FEA), chlorogenic acid (CHA), neochlorogenic acid (NCHA), p–coumaric acid (PCA) and sinapic acid (SA)). The amounts of each detected acid are presented in mg kg^−1^ in Table 5.

The derivatives of benzoic acids (DBA) were present in the samples of the selected *Sorbus* cultivars in smaller amounts, ranging from 56.8 mg kg^−1^ in ‘Discolor’ to 154.7 mg kg^−1^ in ‘Granatina’. The higher amount of 151.4 mg kg^−1^ was also determined in ‘Alaja Krupnaja’.

This study identified significant differences in the presence of individual phenolic acids belonging to the group of derivatives of benzoic acid. For example, gallic acid (GA) was recorded ranging from 1.6 mg kg^−1^ in ‘Discolor’ and ‘Titan’ to 16.7 mg kg^−1^ in ‘Koncentra’. Gallic acid was not detected in the samples of *Sorbus umbellata* from Turkey [24]. As the prevailing acid from the group of benzoic acid derivatives, vanillic acid (VA) was measured in the amount of 37.8 mg kg^−1^ only in the light-colored cultivar ‘Alaja Krupnaja’. Kıvrak et al. (2014) confirmed the presence of this acid in a lower amount (2.52 mg. kg^−1^) in *Sorbus umbellata* fruits [24].

Syringic acid (SI) content varied significantly, ranging between 0.6 mg kg^−1^ in ‘Titan’ and 44.3 mg kg^−1^ in ‘Granatina’. In ‘Discolor’, it was detected in the amount of 2.4 mg kg^−1^, which is in accordance with the 2.91 mg kg^−1^ found in fruits of *Sorbus umbellata* from Turkey by Kıvrak et al. (2014) [24]. In the majority of the samples, protocatechuic acid (PA) was the most prominent derivative of benzoic acid, with contents ranging from 11.9 mg kg^−1^ in ‘Discolor’ to 52.1 mg kg^−1^ in ‘Granatina’. PA was also identified in the fruits and leaves of *Sorbus aucuparia* by Olsewska et al. (2012) [9].

Even though Kıvrak et al. (2014) established protocatechuic acid ethyl ester (PCEE) in a concentration of 74.45 mg kg^−1^ in *Sorbus umbellata* fruits from Turkey, it was detected in lower amounts (ranging from 0.7 mg kg^−1^ in ‘Koncentra’ to 9.0 mg kg^−1^ in ‘Titan’) in this study [24].

Furthermore, hydroxybenzoic acid (HB) showed a great variability in its contents, ranging from 2.6 mg kg^−1^ in ‘Koncentra’ to 41.0 mg kg^−1^ in ‘Alaja Krupnaja’. Kıvrak et al. (2014) declared even lower amounts (1.41 mg kg^−1^) in *Sorbus umbellata* fruits from Turkey [24]. In all the cultivars examined in this study, ellagic acid was detected in a range from 0.9 mg kg^−1^ in ‘Alaja Krupnaja’ to 31.6 mg kg^−1^ in ‘Discolor’.

When compared with benzoic acid derivatives, the derivatives of cinnamic acid (DCA) were present in higher amounts, ranging from 467.9 mg kg^−1^ in ‘Discolor’ to 6847.1 mg kg^−1^ in ‘Granatina’. Considerable amounts were also observed in the cultivars ‘Granatnaja’ and ‘Titan’, in amounts of 6292.4 mg kg^−1^ and 6265.1 mg kg^−1^, respectively.

Chlorogenic (3-*O*-caffeoylquinic acid, 3–CQA) (CHA) and neochlorogenic (5-*O*-caffeoylquinic acid, 5–CQA) (NCHA) acids were the main phenolic acids in sweet rowanberry, which is in alignment with the results of this study [10,21]. Both of these acids are considered markers of the phytochemical and antioxidant profiles of *Sorbus* fruits and were present in all the samples of the analyzed *Sorbus* cultivars [29].

Chlorogenic acid was determined to be the most abundant phenolic compound in *S. aucuparia* fruits [36] with its content of 200 mg 100 g^−1^ [3] which is in accordance with this study. The content of CHA ranged from 189.7 mg kg^−1^ in ‘Discolor’ to 2375.2 mg kg^−1^ in ‘Titan’. A high amount of CHA was also detected in ‘Koncentra’ and ‘Granatina’, with values of 2277.4 mg kg^−1^ and 2271.9 mg kg^−1^, respectively.

Neochlorogenic acid was established in a wide range, from 43.6 mg kg^−1^ in ‘Discolor’ to 4069.8 mg kg^−1^ in ‘Granatina’. Bobinaitė et al. (2020) identified the highest contents of neochlorogenic acid in the cultivars ‘Likernaja’ and ‘Solnechnaja’, and reasonably high contents were also determined in ‘Burka’, ‘Bussinka’ and ‘Granatnaja’ [37]. The content of these caffeoylquinic acids in the tested cultivars by Zymone et al. (2018) varied significantly, up to 16-fold [26].

Jurikova et al. (2014) determined the highest contents of chlorogenic acid in ‘Likernaja’ (100.9 mg 100 g^−1^ FW) and ‘Granatnaja’ (90.62 mg 100 g^−1^ FW) [12]. Similarly, high amounts of NCHA and CHA were established by Raudonis et al. (2014) in the cultivars ‘Koncentra’ and ‘Granatina’ from Lithuania, with values of 1608 mg kg^−1^ and 1220 mg kg^−1^, respectively [29]. Chlorogenic acid was the most abundant cinnamic acid in ‘Granatnaja’ with the amount of 906.2 mg kg^−1^ FW harvested in the Czech Republic in 2012 and 2013 [12]. A great diversity in NCHA and CHA contents was found in 26 cultivars of sweet rowanberry from Serbia and Montenegro; NCHA ranged between 720 mg kg^−1^ and 7030 mg kg^−1^, and CHA between 350 mg kg^−1^ and 10,010 mg kg^−1^ [10]. In contrast, in two cultivars from Finland, lower contents were detected (CHA between 534 mg kg^−1^ FW and 747 mg kg^−1^ in ‘Granatnaja’, and NCHA ranging from 479 mg kg^−1^ FW to 692 mg kg^−1^ FW in ‘Titan’) [3]. Another low CHA content of 7.51 mg kg^−1^ was reported in *Sorbus umbellata* fruits from Turkey [24]. Cultivars from Lithuania contained lower NCHA amounts, specifically ‘Granatnaja’ with 2555 mg kg^−1^, ‘Businka’ with 1722 mg kg^−1^, ‘Koncentra’ with 820 mg kg^−1^ and ‘Titan’ with 2347 mg kg^−1^; the cultivar ‘Alaja Krupnaja’, with a content of 1588 mg kg^−1^ FW, was the exception. CHA content was reported to be higher in the cultivars ‘Granatnaja’, with an amount of 2425 mg kg^−1^, ‘Businka’, with 3130 mg kg^−1^, and ‘Titan’, with 2530 mg kg^−1^, compared to cultivars with lower CHA amounts: ‘Alaja Krupnaja’, with an amount of 1191 mg kg^−1^, and ‘Koncentra’, with 1804 mg kg^−1^ [26].

Coffeic acid (CA) dominated in ‘Alaja Krupnaja’ with a value of 1803.6 mg kg^−1^; in the rest of the samples, it ranged from 51.8 mg kg^−1^ in ‘Titan’ to 667.4 mg kg^−1^ in ‘Granatnaja’. Kivrak et al. (2014) recorded only an insignificant content of coffeic acid with a value of 3.03 mg kg^−1^ in the samples from Turkey [24].

The highest content of FEA in the amount of 115.8 mg kg^−1^ was present in ‘Alaja Krupnaja’. Additionally, it ranged from 4.3 mg kg^−1^ in ‘Titan’ to 9.1 mg kg^−1^ in ‘Granatina’. However, it was not detected in the cultivar ‘Discolor’. Such low values are in accordance with the amount of 7.67 mg kg^−1^ detected in the samples of *Sorbus umbellata* from Turkey [24].

P– coumaric acid (PC) was present only in low amounts, ranging from 2.4 mg kg^−1^ in ‘Alaja Krupnaja’ to 13.6 mg kg^−1^ in ‘Titan’. It was not detected in *Sorbus umbellata* fruits [24]. Syringic acid (SA) ranged from 4.1 mg kg^−1^ in ‘Titan’ to 61.8 mg kg^−1^ in ‘Granatina’. Hydroxycinnanic acid (HCA) was not detected, and t–cinnamic acid (TCA) was recorded only in a trace amount of 0.9 mg kg^−1^ in ‘Alaja Krupnaja’.

### 3.4. Influence of Individual Phenolic Compounds on Total Phenolic Content (TPC), Total Flavonoid Content (TFC) and Anthocyanin Content (AC)

The polyphenolic substances mainly responsible for the antioxidant properties of *Sorbus* fruits include phenolic acids (predominantly caffeoylquinic acids), flavonols (represented by quercetin, isoquercetin, hyperoside, rutin, catechin and epicatechin), anthocyanins (primarily cyanidin or pelargonidin glycosides) and proanthocyanidins [4].

This study establishes the correlations between total phenolic content (TPC), total flavonoid content (TFC), total anthocyanin content (AC) and individual phenolic compounds, with the values of these correlation coefficients displayed in Table 6 and Table 7.

As Table 6 shows, the relation between TPC and TFC can be evaluated as a very weak positive linear correlation (R = 0.0293).

It has been found that the rise in anthocyanin content in the cultivated species does not correspond with enhanced antioxidant activity [8]. Similarly, this study has shown a linear correlation between TPC and AC of R = −0.4034.

Regarding the relation between RU and TPC, a linear correlation with the highest value of R = 0.4853 was established. Weak linear correlations between the contents of individual flavanols (EGC, EC and C), the total content of flavanals (FLAVAN) and TPC were analyzed. Nevertheless, a stronger positive linear correlation of R = 0.5324 was established between stilbene RES and TPC.

Between TFC and RU, a direct linear correlation of R = 0.2540 was verified. Regarding the group of individual flavonols, EC had the most significant impact, corresponding with the highest value of R = 0.5484. Between TFC and EGC, only an indirect negative correlation of R = −0.4116 was established. Flavanals showed a less significant impact on TFC, with a negative correlation value of R = −0.1054.

An indirect linear correlation between AC and RU with a negative value of R = −0.3693 was identified; furthermore, a very strong linear correlation between AC and catechins was established (R = 0.6988). The correlations between AC and catechins were very weak (R = 0.0217 for EC and R = −0.1275 for EGC). The correlation between flavanols (FLAVAN) and AC can be evaluated as a linear direct correlation with R = 0.2776.

As is evident from Table 7, the phenolic acids from the DBA group influenced TPC more than the acids from the DCA group. A direct linear correlation between TPC and DBA was established (R = 0.9990). On the other hand, DCA displayed only a weak indirect linear correlation of R = −0.1457. Regarding DBA, the strongest correlations were identified for HB (R = 0.5977) and SI (R = 0.5514); VA, PC and PCEE displayed weaker direct correlations of R = 0.3074, R = 0.1356 and R = 0.2504, respectively. Finally, GA and EL showed very weak indirect correlations of R = −0.1277 and R = −0.0218, respectively.

In the DCA group, PCA displayed the strongest indirect correlation of R = −0.5892. Furthermore, CHA and NCHA showed weaker negative correlations (R = −0.2836 and R = −0.2543, respectively). Positive linear correlations were identified between TPC and CA (R = 0.4222), between TPC and FEA (R = 0.4735) and, lastly, between TPC and SA (R = 0.3555).

### 3.5. Determination of Vitamin C and E

The values of vitamins C and E in the lyophilized samples of the selected *Sorbus* cultivars are provided in Table 8.

Paulovicsova et al. (2009) recorded 22.84 ± 1 mg 100 g^−1^ of ascorbic acid in the sweet rowanberry ‘Moravska Sladkoploda’, cultivated in Slovakia [25].

The content of ascorbic acid in the samples of sweet rowanberry cultivars was monitored in the amount of 12–21 mg 100 g^−1^ in ‘Granatnaya’ and 86 mg 100 g^−1^ in ‘Zholtaya’ [38,39]. The study by Ozolina and Kampuse (2019) showed the content of vitamin C in the sweet rowanberry juice residues in the amounts of 60.56 ± 5.33 mg 100g^−1^ [40].

As can be seen from the results in Table 8, vitamin C contents showed statistical differences between the cultivars, ranging between 4.87 g kg^−1^ in ‘Titan’ and 7.9 g kg^−1^ in ‘Alaja Krupnaja’. The average reported vitamin C content in sweet rowanberries reached a value of 4.85 g kg^−1^ FW, which is comparable with its content in ‘Titan’ [40].

Lower values of ascorbic acid were found in the samples of two cultivars of sweet rowanberry, ‘Granatina’, with a value of 2.10 g kg^−1^ FW, and ‘Titan’, with 1.51 g kg^−1^ FW, both from the Czech Republic [6], and in the samples of ‘Alaja Krupnaja’ (0.24 g kg^−1^), ‘Granatnaja’ (0.20 g kg^−1^), ‘Koncentra’ (0.36 g kg^−1^) and ‘Titan’ (0.20 g kg^−1^) from Lithuania. In ‘Businka’, ascorbic acid was not detected [26], and a low value of 0.68 g kg^−1^ FW was determined in the samples from Poland [22].

Generally, vitamin E has been recorded in lower amounts than vitamin C. The lowest vitamin E content of 1.42 mg kg^−1^ was detected in the cultivar ‘Alaja Krupnaja’. Otherwise, its content ranged from 3.96 mg kg^−1^ in ‘Titan’ to 4.77 mg kg^−1^ in ‘Businka’.

### 3.6. Antioxidant Activity by DPPH, ACW and ACL

The values of DPPH, ACW and ACL in the lyophilized samples of the selected *Sorbus* cultivars are presented in Table 9.

As Table 9 shows, the antioxidant activity determined by DPPH displayed lower values in comparison with the results obtained by ACW and ACL. What is more, statistically significant differences between the cultivars were established in nearly all samples. DPPH values ranged from 3.32 g Trolox kg^−1^ in ‘Discolor’ to 16.16 g Trolox kg^−1^ in ‘Businka’. The DPPH radical-scavenging activity of the evaluated samples from Latvia reported by Kampuss et al. (2008) ranged from 2.5 g to 11.2 g per g of DPPH radical determined in ‘Alaja Krupnaja’ and ‘Likernaja’, respectively [27].

Lower values of DPPH were reported in three cultivars from the Czech Republic in 2011 and 2012 (‘Granatnaja’, with a value of 9.50 g AK kg^−1^ FW, ‘Granatina’, with 9.62 g AK kg^−1^ FW, and ‘Titan’, with 7.39 g AK kg^−1^ FW [6]) and two Czech cultivars harvested in 2012 and 2013 (‘Granatnaja’, with a value of 4.8 g AK kg^−1^ FW, and ‘Granatina’, with 5.8 g AK kg^−1^ FW [12]).

Compared with the other methods, the ACW method provided the most significant values of antioxidant activity, with the highest record of 156.87 g AK kg^−1^ in ‘Alaja Krupnaja’. The lowest values of 61.70 g AK kg^−1^ and 63.59 g AK kg^−1^ were detected in ‘Discolor ‘and ‘Titan’, respectively.

Regarding ACL, the values were significantly lower than those obtained by ACW. They ranged from 15.11 g Trolox kg^−1^ in ‘Alaja Krupnaja’ and 15.90 g Trolox kg^−1^ in ‘Titan’ to 23.32 g Trolox kg^−1^ in ‘Granatina’ and 22.11 g Trolox kg^−1^ in ‘Koncentra’.

The antioxidant activity values differ significantly in the published data as well, reflecting the influence of the applied method. For example, samples of sweet rowanberry from Poland showed antioxidant activity values of 10.75 μmol g^−1^ FW after using FRAP; however, only 5.94 μmol Trolox g^−1^ FW when ABTS radicals were employed [22]. A high AOA value detected by DPPH of 62.09 µg mL^−1^ was reported in the fruit of *Sorbus torminalis* (L.) Crantz from Turkey by Kıvrak et al. (2014) [24]. Another study emphasized the significant influence of the extraction solution on the final AOA value detected by DPPH; it reached 32.31 mg mL^−1^ after usage of the methanolic extract solution contrasting to 5.69 mg mL^−1^ after the application of the water extraction [41].

### 3.7. Influence of Various Factors on Antioxidant Activity

The method of regression analysis was applied to determine the correlation between the results for AOA, which were analyzed using three different methods: DPPH, ACW and ACL. The influence of TPC, TFC, AC and vitamins C and E on the AOA values was examined as well. The correlation coefficients are provided in Table 10.

#### 3.7.1. Influence of the Method of Antioxidant Activity (AOA) Detection

The relation between AOA determined by the DPPH method and TPC differs between studies. Kampuss et al. (2008) determined a considerable positive correlation only between the antioxidant activity and TPC (R = 0.886) [27]. In contrast with the results of this study, a negative correlation between AOA and TPC was reported in other studies [3,42].

This study showed a weak correlation between three methods of AOA detection: a positive direct correlation between DPPH and ACW (R = 0.3987), a very low correlation between DPPH and ACL (R = 0.0232) and an indirect correlation between ACW and ACL (R = −0.1965). A positive correlation of R = 0.977 was reported between DPPH and ABTS in the samples of hybrids of sweet rowanberry from Turkey, and indirect correlations between FRAP and DPPH (R = −0.703) and between FRAP and ABTS (R = −0.837) [41].

#### 3.7.2. Influence of Total Phenolic Content (TPC)

Regarding the relation between TPC and ACW, a direct linear correlation with the highest value of R = 0.7671 was detected. Between TPC and DPPH, a very low correlation (R = 0.0596) was identified. Furthermore, the relation between TPC and ACL responded with an indirect, very low correlation of R = −0.2162. In the cultivars of sweet rowanberry from the Czech Republic harvested in 2011 and 2012, a high value of correlation between TPC and DPPH (R = 0.8904) was recorded [6]. These differences in correlations within phenolic groups and methods of AOA determination, despite using the same extracts, may be explained by different reaction mechanisms in the ORAC, ABTS^●+^ and DPPH^●^ analyses [21].

The AOA values of the fruit extracts of *S. torminalis* were significantly influenced by the total phenolic content (TPC) established by the Folin–Ciocalteu method [9].

Notably variable correlations were reported between TPC and different methods for AOA detection in fruits of sweet rowanberry from Turkey: an indirect correlation between TPC and DPPH with a value of R = −0.728 and between TPC and ABTS with R = −0.855, contrasting with the positive linear correlation (R = 0.999) monitored between TPC and FRAP [41]. Similarly, in *Sorbus* samples from Poland, correlations between TPC and FRAP with a value of R = 0.993 and between TPC and ABTS with R = 0.943 were established [22]. In the six cultivars of *Sorbus* from Finland, a positive correlation (R = 0.868) was reported between TPC and FRAP, contrasting with the indirect correlation (R = −0.893) between TPC and DPPH [3].

#### 3.7.3. Influence of Total Flavonoid Content (TFC)

The values of AOA determined by DPPH, ACW and ACL in the examined cultivars correlated with TFC, showing different trends. A low direct correlation between DPPH and FL (R = 0.3279) was determined, compared to the value of R = 0.8345 detected in the samples from the Czech Republic published by Mlček et al. (2014) [6]. Correspondingly, a considerable correlation between TFC and AOA in *Sorbus* fruits was observed by Kähkönen et al. (2001) [43].

A correlation between PCL and ACL (R = 0.4488) was established in contrast to the very low negative correlation between ACL and TFC.

#### 3.7.4. Influence of Anthocyanin Content (AC)

The increased anthocyanin content in the cultivated species caused an insignificant rise in antioxidant activity [8].

The values of AOA detected by DPPH, ACW and ACL correlated with AC in various ways. A strong linear correlation between DPPH and AC was established, with R = 0.7132 representing the highest recorded value. Between AC and ACW and also AC and ACL, indirect negative correlations of R = −0.2227 and R = −0.0214 were determined, respectively. A high diversity between AC and FRAP/DPPH was published in the samples from Finland: between FRAP and AC with a value of R = 0.470 and between DPPH and AC with a value of R = −0.165 [3].

#### 3.7.5. Influence of Vitamins C and E

A very strong linear correlation of R = 0.9024 was established between vitamin C and ACW and a weaker correlation of R = 0.2399 between vitamin C and DPPH. Jabłońska–Ryś et al. (2009) detected stronger relations between vitamin C and FRAP (R = 0.984) and ABTS (R = 0.925) in the samples from Poland [22]. Between vitamin C and ACL, an indirect linear correlation of R = −0.0789 was detected. Between vitamin E and ACW, an indirect weak correlation of R = −0.5352 was established.

A correlation between ascorbic acid and TAC (R = 0.9312) was recorded by Mlcek et al. (2014) when examining *Sorbus* cultivars from the Czech Republic in 2011 and 2012 [6].

#### 3.7.6. Influence of Flavonols and Flavanols

Statistically significant correlations were reported between total flavonol content and antioxidant activity in sweet rowanberry cultivars from Finland [43].

Table 11 shows the values of the correlations between DPPH, ACW, ACL and the content of the flavonol rutin (RU), and the individual flavanols EGC, EC and C and total flavanols (FLAVAN) and stilbene resveratrol (RES). It is evident that flavanol catechin (C) mainly contributed to AOA as determined by the DPPH method (R = 0.8624). A direct linear correlation was estimated for epigallocatechin (EGC) with R = 0.2852 and for total flavanols (FLAVAN) with R = 0.6155. Epigallocatechin (EGC) and flavonol rutin (RU) displayed an indirect correlation with negative correlation coefficients of R = −0.4512 and R = −0.2054, respectively.

Various correlations were identified with regard to the AOA detected by PCL. Between RU and ACW, a direct correlation of R = 0.6032 was determined contrasting with a weak indirect correlation between RU and ACL with R = −0.0595. Flavanols EC and ACW displayed an indirect correlation of R = −0.6050, in contrast to flavanols EGC, C and total flavanols (FLAVAN), which showed a weak direct correlation. The relation between ACL and individual flavanols showed direct linear correlations (R = 0.4562 for EGC, R = 0.2042 for EC and R = 0.3741 for K). The correlation between ACL and total flavanols (FLAVAN) was determined at R = 0.4885.

Between stilbene RES and ACW, a very strong linear correlation of R = 0.7169 was established, contrasting with the negative correlation of R = −0.6560 determined between RES and ACL. A very weak indirect correlation was detected between RES and DPPH (R = −0.1611).

#### 3.7.7. Influence of Phenolic Acids

Mattila et al. (2006) discovered that, primarily, phenolic acids were responsible for antioxidant activity in sweet rowanberry fruits [11]. Furthermore, neochlorogenic and chlorogenic acids were determined as its markers [29].

Regarding phenolic acids, a considerable positive correlation was established, especially between hydroxycinnamic acid and antioxidant activity, in the samples of interspecific hybrids of sweet rowanberry from Finland [43].

Correlations between DPPH, ACW and ACL with the content of individual phenolic acids (GA, VA, SI, PK, PKEE, HB, EL, KA, FER, CHL, KU and SP) as well as with the total content of benzoic acid derivatives (DBA) and cinnamic acid derivatives (DCA) were determined by regression analyses. Table 12 provides their R values.

As can be seen, DCA contributed to the AOA detected by DPPH to a greater extent (R = 0.7865) than DBA did (R = 0.4083). On the other hand, Sarv et al. (2021) identified weak correlations between different methods of AOA determination, including ORAC, ABTS^●+^ and DPPH, and HCA contents in the pomace extracts from sweet rowanberry [21].

DBA and ACW displayed a higher correlation value of R = 0.8478 than ACW and DCA (R = 0.3289). What is more, DBA and ACL showed a very weak indirect correlation (R = −0.0392) and DCA and ACL showed a very weak linear correlation (R = 0.0877). High variability was also reported in the correlations between DCA and FRAP (R = 0.070) and DPPH (R = −0.205) in the samples from Finland [3].

Regarding individual phenolic acids from the DBA group and DPPH, direct linear correlations were established (except for EL with R = −0.4118). The highest values of correlation coefficients were determined between DPPH and the acids PK and SI, with R = 0.6812 and R = 0.5412, respectively.

The highest value of R = 0.8014 displayed a correlation between DPPH and NCHA, followed by CHA (R = 0.6697). SP, with a value of R = 0.5326, showed a positive correlation with DPPH as well. PCA proved a very weak correlation (R = 0.0230). FEA displayed an indirect correlation with DPPH (R = −0.5284) and a very weak indirect correlation with CA (R = −0.0403).

Similarly, individual phenolic acids belonging to the DBA group and ACW showed direct correlations (except for EL with R = −0.4447). The highest values of the correlation coefficients of R = 0.8541, R = 0.6681 and R = 0.5314 were detected between ACW and HB, SI and PK acids, respectively. With regard to the acids belonging to the DCA group, the strongest linear correlations were determined between ACW and CA and FEA, with values of R = 0.7160 and R = 0.7032, respectively. The rest of the phenolic acids from the DCA group (CHA, NCHA and SA) showed only weak linear correlations, and PCA displayed an indirect negative correlation (R = −0.5179).

Regarding the relations between ACL and individual DBA acids, direct linear correlations were determined with these four acids: the strongest correlation was displayed between ACL and GA and VA (R = 0.6063 and R = 0.5961, respectively), EL and SI showed only weaker correlations with R = 0.4468 and R = 0.3461, respectively. An indirect linear correlation was established between ACL and PCEE, with the highest negative value of R = −0.6782, and a weaker correlation between ACL and HB and PC, with R = −0.2809 and R = −0.1143, respectively. Individual phenolic acids from the DCA group and ACL showed the strongest correlation (R = 0.5476). NCHA and CHA with ACL performed weak linear correlations, with values of R = 0.2026 and R = 0.1845, respectively. FEA, CA and PCA provided indirect linear correlations of R = −0.5167, R = −0.3978, and R = −0.0912, respectively.

Strong positive correlations were established between the concentrations of phenolics (specifically proanthocyanidins, caffeoylquinic acids and flavonoid aglycones) and the antioxidant properties [3,10,16,23].

Šavikin (2017) compared the chemical composition of fruits of *S. aria* and *S. aucuparia* from various altitudes. Even though no correlation was detected between TPC, total proanthocyanidins, antioxidant activity and the growing site [10], notable TPC values were confirmed in *S. aucuparia* and higher proanthocyanidin content in *S. aria* [10].

## 4. Conclusions

This paper has examined seven *Sorbus* cultivars and established their total polyphenolic, flavonoid and anthocyanin contents, as well as the contents of individual polyphenolic compounds belonging to the group of phenolic acids and flavonoids. Additionally, it has determined their antioxidant activity by employing various methods and assessed the relations and mutual influences of these compounds on the resulting antioxidant activity, also showing the impact of the method used through the evaluation of the correlation coefficients.

The highest total phenolic content of 8307.4 mg kg^−1^ was recorded in ‘Granatina’ with the main contribution being provided by phenolic acid content (7001.7 mg kg^−1^) and total flavonoid content (1304.6 mg kg^−1^). The lowest total phenolic content of 857.4 mg kg^−1^ was recorded in ‘Discolor’. Flavanols represented the most abundant group of flavonoids in *Sorbus* fruits; catechin was the second most frequent flavanol, with the highest content of 633.67 mg g^−1^ in ‘Granatina’. Flavonols were represented by rutin and quercetin. ‘Businka’ displayed a significant vitamin E content of 4.77 mg kg^−1^, and ‘Alaja Krupnaja’ had the highest vitamin C level (7.89 g kg^−1^).

These results have proven the valuable potential health and nutritional benefits of *Sorbus* fresh fruits and their promising role in the food processing industry. What is more, this study facilitates the selection of the most suitable cultivars for producers and consumers based on a diverse array of aspects.

## Figures and Tables

**Table 1:** Characteristics of the selected *Sorbus* cultivars.

Cultivar	Origin/Breeding Background	Description
‘Alaja Krupnaja’	Russia *S. aucuparia* × *Pyrus* sp. × *S. aucuparia* var. *moravic*	Round, bright red-brown colored fruits with mildly bitter taste, 0.6 g, August–September
‘Granatnaja’	Russia *S. aucuparia* × *Crataegus sanguinea* = *Sorbocrataegus miczurinii*	Round, dark red or brown fruits with sweet and sour taste, 1.7 g, August–September
‘Granatina’	Slovakia *S. aucuparia* × *Crataegus sanguinea* × *Crateagus laevigata*	Dark red fruit of medium size, sweet–sour taste, 1.2 g, August–September
‘Businka’	Russia Seedling of *Kubovaja* (*S. aucuparia*)	Large, long fruit (1 cm, 1.5 g) yellow-red or dark red color with a taste reminiscent of blueberry, September–October
‘Discolor’	China	Round fruit with a color varying from red to creamy yellow turning to pink, Ø 0.7–1 cm, 3 g, sweet–sour taste, September–October
‘Titan’	Russia *‘*Burka’ × (*Sorbus aucuparia*) × (*Malus* sp.) × (*Pyrus* sp.)	Round-shaped fruit, dark red or violet, without typical aroma, 2 g, suitable for processing, September–October
‘Koncentra’	Germany	Round-shaped fruit, orange, with a sour taste due to the significant content of vitamin C, September–October

**Table 2:** Climatic characteristics of the experimental area in Žabčice.

Žabčice	Long-Term Average	2014
Temperature [°C]	9.2	11.2
Precipitation [mm]	480	577

**Table 3:** Total phenolic content (TPC) [g GA kg^−1^], total flavonoid content (TFC) [g Trolox kg^−1^] and anthocyanin content (AC) [mg COG 100 g^−1^] in the selected *Sorbus* cultivars.

*Sorbus*—Cultivars	Total Phenolic Content (TPC) [g GA kg^−1^]		Total Flavonoid Content (TFC) [g RU kg^−1^]		Anthocyanins (AC) [mg COG 100 g^−1^]	
	Mean	SD	Mean	SD	Mean	SD
‘Alaja Krupnaja’	15.66 ^a^	0.16	17.28 ^a^	0.49	1.19 ^a^	0.18
‘Granatnaja’	8.81 ^b^	0.09	26.69 ^b^	0.12	51.38 ^b^	0.16
‘Granatina’	14.75 ^c^	0.08	26.85 ^b^	0.17	32.52 ^c^	1.02
‘Businka’	16.31 ^d^	0.02	21.22 ^c^	0.06	36.35 ^d^	1.44
‘Discolor’	12.63 ^e^	0.02	22.81 ^d^	0.06	5.50 ^e^	0.08
‘Koncentra’	10.56 ^f^	0.02	15.04 ^e^	0.11	5.36 ^f^	0.18
‘Titan’	9.00 ^b^	0.02	18.29 ^f^	0.08	50.20 ^g^	1.04

The results are expressed as arithmetic means ± SD (n = 6). The values in a row with different superscripts indicate a statistically significant difference at the significance level of *p* < 0.05.

**Table 4:** Total flavonoid content and contents of individual flavonoids and stilbenes [mg kg^−1^] in the selected *Sorbus* cultivars.

Flavonoids [mg kg^−1^]	*Sorbus*—Cultivars													
	‘A. Krupnaja’		‘Granatnaja’		‘Granatina’		‘Businka’		‘Discolor’		‘Koncentra’		‘Titan’	
	Mean	SD	Mean	SD	Mean	SD	Mean	SD	Mean	SD	Mean	SD	Mean	SD
*Flavonols*														
quercetin	2.4 ^a^	0.0	nd		nd		nd		nd		nd		nd	
rutin	71.1 ^a^	0.3	33.1 ^b^	0.3	50.8 ^c^	0.6	13.7 ^d^	1.5	33.9 ^e^	0.0	9.8 ^f^	0.3	10.6 ^f^	0.9
kaempferol	nd		nd		nd		nd		nd		nd		nd	
*Flavanols*														
epigallocatechin	423.4 ^a^	0.4	533.1 ^b^	0.5	610.0 ^c^	9.0	625.4 ^d^	1.8	244.3 ^e^	8.1	1167.5 ^f^	4.6	458.4 ^g^	6.4
epicatechin	4.2 ^a^	0.2	17.7 ^b^	1.5	10.3 ^c^	0.0	6.8 ^d^	0.1	31.1 ^e^	0.0	3.3 ^f^	0.1	9.6 ^g^	0.2
catechin	165.5 ^a^	3.2	583.1 ^b^	6.0	633.6 ^c^	1.9	560.5 ^d^	4.9	23.4 ^e^	0.1	497.7 ^f^	1.2	475.6 ^g^	0.8
Stilbenes														
resveratrol	3.3 ^a^	0.1	0.9 ^b^	0.0	1.1 ^c^	0.0	0.8 ^d^	0.0	nd		0.8 ^d^	0.0	0.5 ^e^	0.0
Total content														
*Flavonols*	73.5 ^a^	0.3	33.1 ^b^	0.3	50.8 ^c^	0.6	13.7 ^d^	1.5	33.9 ^e^	0.0	9.8 ^f^	0.3	10.6 ^f^	0.9
*Flavanols*	593.1 ^a^	3.7	1135.4 ^b^	8.0	1253.8 ^c^	10.9	1192.7 ^d^	6.9	298.8 ^e^	8.2	1668.6 ^f^	5.8	943.6 ^g^	7.4

nd—not detected. The results are expressed as arithmetic means ± SD (n = 6). The values in a row with different superscripts indicate a statistically significant difference at the significance level of *p* < 0.05.

**Table 5:** Total phenolic acid content and contents of individual phenolic acids [mg kg^−1^] in the selected *Sorbus* cultivars.

Phenolic acids [mg kg^−1^]	*Sorbus*—Cultivars													
	‘A. Krupnaja’		‘Granatnaja’		‘Granatina’		‘Businka’		‘Discolor’		‘Koncentra’		‘Titan’	
	Mean	SD	Mean	SD	Mean	SD	Mean	SD	Mean	SD	Mean	SD	Mean	SD
*Derivates of benzoic acid*														
gallic	2.6 ^a^	0.0	6.2 ^b^	0.1	7.2 ^c^	0.1	6.5 ^d^	0.0	1.6 ^e^	0.0	16.7 ^f^	0.2	1.6 ^e^	0.0
vanillic	37.8 ^a^	0.3	nd		nd		nd		nd		nd		nd	
syringic	18.6 ^a^	0.5	19.5 ^a^	0.4	44.3 ^b^	0.2	22.7 ^c^	0.2	2.4 ^d^	0.2	3.9 ^e^	0.1	0.6 ^f^	0.0
protocatechuic	44.3 ^a^	0.1	39.3 ^b^	0.8	52.1 ^c^	0.2	41.1 ^b^	0.2	11.9 ^d^	0.0	32.8 ^e^	0.1	47.0 ^f^	0.4
protocatechuic ethyl ester	6.3 ^a^	0.1	4.4 ^b^	0.2	5.5 ^c^	0.1	8.4 ^d^	0.1	2.4 ^e^	0.0	0.7 ^f^	0.1	9.0 ^g^	0.0
4–hydroxybenzoic	41.0 ^a^	0.3	22.1 ^b^	0.1	28.7 ^c^	0.5	21.5 ^d^	0.0	6.9 ^e^	0.0	2.6 ^f^	0.1	9.6 ^g^	0.1
ellagic	0.9 ^a^	0.1	14.8 ^b^	0.2	16.8 ^c^	0.0	6.4 ^d^	0.6	31.6 ^e^	0.2	3.1 ^f^	0.0	2.3 ^g^	0.0
*Derivates of cinnamic acid*														
t–cinnamic	0.9 ^a^	0.0	nd		nd		nd		nd		nd		nd	
hydroxycinnamic	nd		nd		nd		nd		nd		nd		nd	
caffeic	1803.6 ^a^	6.6	667.4 ^b^	3.3	428.6 ^c^	9.5	275.4 ^d^	0.8	226.7 ^e^	8.2	56.1 ^f^	0.1	51.8 ^g^	1.3
ferullic	115.8 ^a^	0.2	7.1 ^b^	0.2	9.1 ^c^	0.9	8.7 ^c,d^	0.4	nd		7.9 ^c,e^	0.3	4.3 ^f^	0.1
chlorogenic	1383.1 ^a^	7.4	1961.8 ^b^	30.0	2271.9 ^c^	25.0	1767.8 ^d^	13.4	189.7 ^e^	5.4	2277.4 ^c^	25.4	2375.2 ^f^	38.6
neochlorogenic	1312.2 ^a^	6.9	3627.5 ^b^	60.7	4069.8 ^c^	11.3	2955.0 ^d^	12.8	43.6 ^e^	0.9	2239.3 ^f^	11.9	3816.0 ^g^	9.4
p–coumaric	2.4 ^a^	0.0	3.9 ^b^	0.1	5.8 ^c^	0.0	2.7 ^d^	0.0	2.8 ^d^	0.1	6.3 ^e^	0.1	13.6 ^f^	0.2
sinapic	7.1 ^a^	0.1	24.8 ^b^	0.1	61.8 ^c^	1.0	26.4 ^d^	0.9	5.2 ^e^	0.1	6.4 ^f^	0.3	4.1 ^g^	0.2
Total content														
*Derivates of benzoic acid*	151.4 ^a^	1.4	106.3 ^b^	1.7	154.7 ^c^	1.1	106.6 ^b^	1.1	56.8 ^d^	0.5	59.7 ^e^	0.5	70.8 ^f^	0.5
*Derivates of cinnamic acid*	4625.1 ^a^	21.2	6292.4 ^b^	94.3	6847.1 ^c^	47.6	5036.2 ^d^	28.3	467.9 ^e^	14.7	4593.4 ^f^	38.1	6265.1 ^b^	48.1

nd—not detected; the results are expressed as arithmetic means ± SD (n = 6). The values in a row with different superscripts indicate a statistically significant difference at the significance level of *p* < 0.05.

**Table 6:** Correlation coefficients (R) between total phenolic content (TPC), total flavonoid content (TFC), total anthocyanin content (AC) and individual phenolic compounds in the selected *Sorbus* cultivars.

	TPC	TFC	AC
TPC	–	0.0293	−0.4034
Flavonols			
RU	0.4853	0.2540	−0.3693
Flavanols			
EGC	−0.1541	−0.4116	−0.1275
EC	−0.2442	0.5484	0.0217
C	−0.1802	0.2846	0.6988
FLAVAN	−0.1964	−0.1054	0.2776
Stilbenes			
RES	0.5324	–	–

*p* < 0.05 significance level.

**Table 7:** Correlation coefficients (R) between total phenolic content (TPC) and individual phenolic acids (GA, VA, SI, PCA, PCEE, HB, EL, HCA, CA, FEA, CHA, NCHA, PKA and SI), total derivatives of benzoic acid (DBA) and total derivatives of cinnamic acid (DCA) in the selected *Sorbus* cultivars.

TPC			
*Derivates of benzoic acid*		*Derivates of cinnamic acid*	
GA	−0.1277	HCA	–
VA	0.3074	CA	0.4222
SI	0.5514	FEA	0.4735
PC	0.1356	CHA	−0.2836
PCEE	0.2504	NCHA	−0.2543
HB	0.5977	PCA	−0.5892
EL	−0.0218	SA	0.3555
DBA	0.990	DCA	−0.1457

*p* < 0.05 significance level.

**Table 8:** The content of vitamins C [g kg^−1^] and E [mg kg^−1^] in the selected *Sorbus* cultivars.

*Sorbus*—Cultivars	Vitamin C [g kg^−1^]		Vitamin E [mg kg^−1^]	
	Mean	SD	Mean	SD
‘Alaja Krupnaja’	7.89 ^a^	0.04	1.42 ^a^	0.01
‘Granatnaja’	6.12 ^b^	0.00	4.13 ^b^	0.01
‘Granatina’	6.41 ^c^	0.01	4.41 ^c^	0.02
‘Businka’	6.72 ^d^	0.01	4.77 ^d^	0.02
‘Discolor’	5.16 ^e^	0.03	4.49 ^e^	0.03
‘Koncentra’	6.85 ^f^	0.01	4.26 ^f^	0.01
‘Titan’	4.87 ^g^	0.10	3.96 ^g^	0.04

The results are expressed as arithmetic means ± SD (n = 6). The values in a row with different superscripts indicate a statistically significant difference at the significance level of *p* < 0.05.

**Table 9:** Antioxidant activity determined by DPPH [g Trolox kg^−1^], ACW [g AK kg^−1^] and ACL [g Trolox kg^−1^] in the selected *Sorbus* cultivars.

*Sorbus*—Cultivars	DPPH [g Trolox kg^−1^]		ACW [g AC kg^−1^]		ACL [g Trolox kg^−1^]	
	Mean	SD	Mean	SD	Mean	SD
‘Alaja Krupnaja’	8.61 ^a^	0.13	156.87 ^a^	0.26	15.11 ^a^	0.26
‘Granatnaja’	14.98 ^b^	0.12	93.35 ^b^	0.63	20.70 ^b^	0.47
‘Granatina’	12.49 ^c^	0.03	124.89 ^c^	0.81	23.32 ^c^	0.53
‘Businka’	16.16 ^d^	0.05	131.67 ^d^	0.64	16.59 ^d^	0.35
‘Discolor’	3.32 ^e^	0.01	61.70 ^e^	0.96	19.62 ^e^	0.23
‘Koncentra’	9.34 ^f^	0.00	92.79 ^b^	0.65	22.11 ^c^	0.73
‘Titan’	10.47 ^g^	0.01	63.59 ^f^	0.58	15.90 ^f^	0.26

The results are expressed as arithmetic means ± SD (n = 6). The values in a row with different superscripts indicate a statistically significant difference at the significance level of *p* < 0.05.

**Table 10:** Correlation coefficients (R) between different methods: DPPH, ACW, ACL, total phenolic content (TPC), total flavonoid content (TFC), anthocyanin content (AC) and the content of vitamins C and E in the samples of the selected *Sorbus* cultivars.

	DPPH	ACW	ACL
DPPH	–		
ACW	0.3987	–	
ACL	0.0232	−0.1965	–
TPC	0.0596	0.7671	−0.2162
TFC	0.3279	−0.0290	0.4488
AC	0.7132	−0.2227	−0.0214
vitamin C	0.2399	0.9024	−0.0789
vitamin E	0.2389	−0.5352	0.5221

*p* < 0.05 significance level.

**Table 11:** Correlation coefficients (R) between different methods for AOA detection (DPPH, ACW and ACL) and individual flavonols (RU), individual flavanols (EGC, EC and C), total flavanols (FLAVAN) and resveratrol (RES) in the samples of the selected *Sorbus* cultivars.

	DPPH	ACW	ACL
*Flavonols*			
RU	−0.2054	0.6032	−0.0595
*Flavanols*			
EGC	0.2852	0.1069	0.4562
EC	−0.4512	−0.6050	0.2042
C	0.8624	0.1369	0.3741
FLAVAN	0.6155	0.1255	0.4885
*Stilbenes*			
RES	−0.1611	0.7169	−0.6560

*p* < 0.05 significance level.

**Table 12:** Correlation coefficients (R) between different methods for AOA detection (DPPH, ACW and ACL) and individual phenolic acids (GA, VA, SI, PC, PCEE, HB, EL, CA, FEA, CHA, NCHA, PCA and SA), as well as total content of derivatives of benzoic acid (DBA) and total content of derivatives of cinnamic acid (DCA) in the samples of the selected *Sorbus* cultivars.

	DPPH	ACW	ACL
*Derivates of benzoic acid*			
GA	0.2357	0.0914	0.6063
VA	0.2893	0.3439	0.5961
SI	0.5412	0.6681	0.3461
PC	0.6812	0.5314	−0.1143
PCEE	0.4838	0.2687	−0.6782
HB	0.3201	0.8541	−0.2809
EL	−0.4118	−0.4447	0.4468
DBA	0.4083	0.8478	−0.0392
*Derivates of cinnamic acid*			
CA	−0.0403	0.7160	−0.3978
FEA	−0.5284	0.7032	−0.5167
CHA	0.6697	0.1488	0.1845
NCHA	0.8014	0.0771	0.2026
PCA	0.0230	−0.5179	−0.0912
SA	0.5326	0.3869	0.5476
DCA	0.7865	0.3289	0.0877

*p* < 0.05 significance level.

## Data Availability

The data presented in this study are available within the article.
